# Olfaction, navigation, and the origin of isocortex

**DOI:** 10.3389/fnins.2015.00402

**Published:** 2015-10-27

**Authors:** Francisco Aboitiz, Juan F. Montiel

**Affiliations:** ^1^Departamento de Psiquiatría, Escuela de Medicina, Centro Interdisciplinario de Neurociencia, Pontificia Universidad Católica de ChileSantiago, Chile; ^2^Facultad de Medicina, Centro de Investigación Biomédica, Universidad Diego PortalesSantiago, Chile; ^3^MRC Functional Genomics Unit, Department of Physiology, Anatomy and Genetics, University of OxfordOxford, UK

**Keywords:** isocortical evolution, plasticity, olfaction, hippocampus

## Abstract

There are remarkable similarities between the brains of mammals and birds in terms of microcircuit architecture, despite obvious differences in gross morphology and development. While in reptiles and birds the most expanding component (the dorsal ventricular ridge) displays an overall nuclear shape and derives from the lateral and ventral pallium, in mammals a dorsal pallial, six-layered isocortex shows the most remarkable elaboration. Regardless of discussions about possible homologies between mammalian and avian brains, a main question remains in explaining the emergence of the mammalian isocortex, because it represents a unique phenotype across amniotes. In this article, we propose that the origin of the isocortex was driven by behavioral adaptations involving olfactory driven goal-directed and navigating behaviors. These adaptations were linked with increasing sensory development, which provided selective pressure for the expansion of the dorsal pallium. The latter appeared as an interface in olfactory-hippocampal networks, contributing somatosensory information for navigating behavior. Sensory input from other modalities like vision and audition were subsequently recruited into this expanding region, contributing to multimodal associative networks.

## Introduction

Mammals are the descendants of one of the two main amniote clades that colonized the ground by developing an egg that could be laid outside of water. This spectacular innovation yielded two lineages that diverged very early and underwent parallel histories since the late Carboniferous period, some 300 million mya. One of these is the stem reptile, giving rise to all known reptiles and birds (together called sauropsids); and the other is represented by the synapsid or mammal-like reptile. At some point in the Cretaceous, both groups underwent a parallel tendency to increase brain size, in the two most successful clades of each branch: mammals and birds. Beside the increase in brain size, both groups developed highly divergent overall brain anatomies. Sauropsids have a nuclear-shaped dorsal ventricular ridge (DVR) and mammals display a six-layered isocortex. Intriguingly, while the gross brain morphology is quite different in these two taxa, studies in the last 50 years have found significant similarities in brain organization and behavior in the two groups (Wang et al., 2010; Ahumada-Galleguillos et al., 2015; Calabrese and Woolley, 2015). The observed disparity between gross morphology and connectivity has raised an agitated controversy regarding the origins and evolution of such patterns (Aboitiz and Montiel, 2007, 2012). Yet, the most important question remains, namely why only mammals evolved a laminated isocortex. In two companion articles to this one (Bosman and Aboitiz, 2015; Montiel and Aboitiz, 2015), as well as in previous articles (Aboitiz et al., 2003; Aboitiz, 2011; Aboitiz and Zamorano, 2013), we have elaborated an evolutionary developmental hypothesis for the unique laminar development and connectivity of the isocortex. Instead, in this paper we will discuss the selective processes that participated in generating these two divergent phenotypes, particularly that of the mammalian isocortex. Basically, we will argue that the observed differences in gross anatomical structures between birds and mammals are the result of contingent adaptations in lifestyle, reflected in the organization of sensory and motor systems, which drove mammalian brain development away from a more conservative developmental trend as is found in sauropsids. Nonetheless, this condition was no obstacle for the parallel development of highly similar functional circuits and behaviors in both lineages (Güntürkün, 2012; Clayton and Emery, 2015).

## The ecological context

The main question we address here is what ecological and adaptive circumstances selected for the origin of the mammalian brain. To answer this, it is necessary to analyze the ecological niche these animals created, and consider the behavioral and sensory adaptations these animals developed. Basically, the special features that characterize the isocortex are the consequence of specific sensory and behavioral adaptations that in turn selected for developmental modulations in the telencephalon. Initially, these modifications included mainly olfaction and touch, which were involved in linear navigation behavior. As mammals invaded new ecological niches, vision and audition provided additional information about distance and location. This resulted in the development of the isocortex as a multimodal associative system that contributed to generate spatial maps of the environment.

### Mesozoic mammals

**Figure 1:**
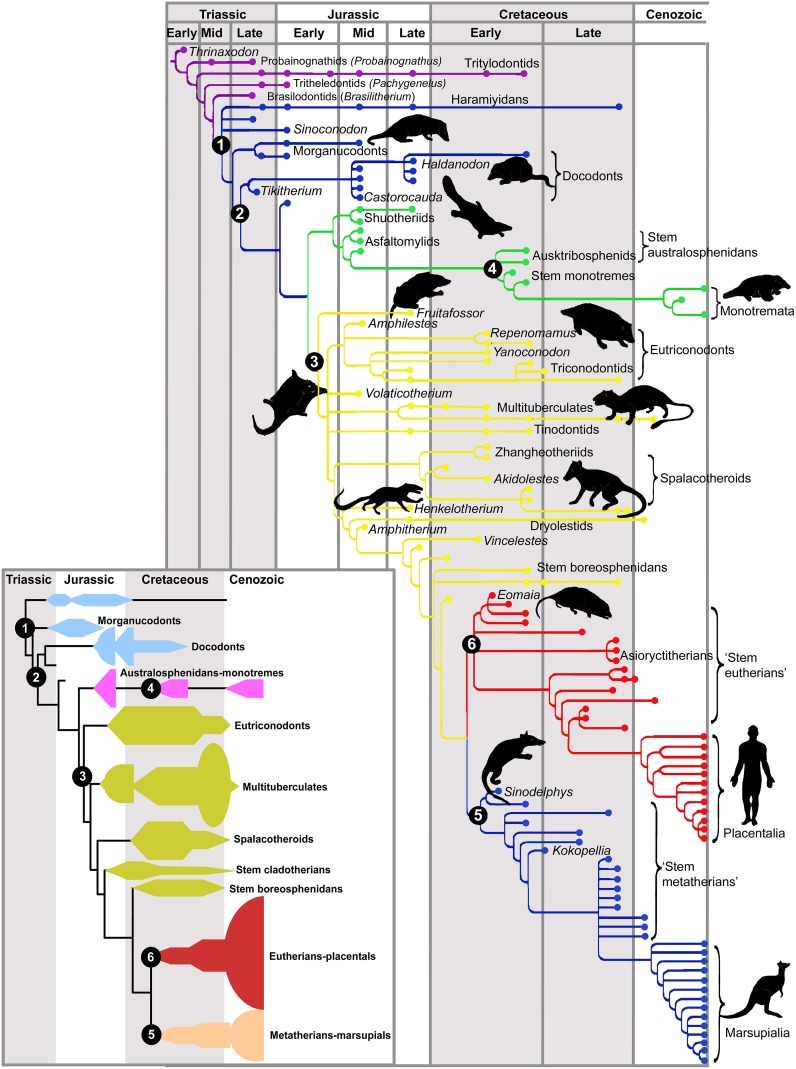
**Phylogeny of fossil mammals**. Critical phylogenetic nodes are shown in numbers. The inset provides a summary diagram of the different clades. Cynodonts are shown in purple. Node (1, blue) mammaliamorphs, a group of advanced cynodonts; (2, also blue) mammaliaforms. The common ancestor of modern mammals (crown mammals) is shown at the base of the green tree. This line gives rise to most mesozoic mammals (3, yellow) and to monotremes (4, green). The common ancestor of therians (marsupials and placentals) stays at the base of (5, blue) marsupials or metatherians and (6, red) placentals or eutherians. Note the dramatic extinction of mammalian lineages at the end of the Cretaceous, concomitant with the extinction of the dinosaurs. With permission from Luo (2007).

Mammals are descendant from cynodonts, a small-sized, omnivorous synapsid lineage originating in the late Permian (about 260 mya), which gave rise to the first mammal-like animals in the mid Triassic (230 mya). The first mammals were a diverse group termed mammaliamorphs (miniaturized mammals such as probainognathids and tritylodontids), which were replaced by mammaliaforms (including moganucodonts and docodonts). Finally, the crown or modern mammals appeared, including fossil triconodontids, multituberculates, and the extant monotremes and therians (marsupials and placentals, or eutherians; Kielan-Jaworowska et al., 2004; Luo, 2007; Rowe and Shepherd, 2015; see Figure 1). The radiation of these early mammalian clades took place quite early and lasted through the Jurassic and Cretaceous, but only the monotremes and therians appear to have lasted beyond the Cretaceous. Thus, contrary to common belief, early mammals underwent a successful adaptive radiation throughout the age of dinosaurs (Luo, 2007).

The origin of mammals was marked by several anatomical and physiological innovations that began with the formation of a secondary palate in cynodonts. This character permitted a separation of the nasal structures from the mouth, generating a moistened air chamber for olfaction. Furthermore, the secondary palate is associated with profound changes in dental structure for mastication, in the tongue for swallowing and in the thoracic cavity that developed a diaphragm for active respiration. The capacity for smell increased, influencing exploratory behavior and providing the possibility for retronasal smell and the generation of complex flavor sensations. These innovations were concomitant with a modest brain expansion in early mammals, as exemplified by *Morganucodon*, a late Triassic-early Jurassic mammaliaform, in which relative brain size was about 50% larger than that of basal cynodonts (Rowe et al., 2011; Rowe and Shepherd, 2015). This increase in brain size is mostly accounted for by an expansion of the olfactory bulb and olfactory cortex, although there is also a larger cortical area and an expanded cerebellum. Moreover, in *Castorocauda*, a mid-Jurassic beaver-tailed mammaliaform, there is evidence of pelt and presumably a somatosensory region in the presumptive isocortex (Rowe et al., 2011). This may have been accompanied by the formation of secretory glands, the production of milk and the acquisition of full homeothermy. An additional increase in brain growth occurred in *Hadrocodium* (a small, early crown mammal some 3 cm long, from the early Jurassic in China), where again most growth was due to the olfactory bulbs and olfactory cortex. In *Hadrocodium*, there is detachment of the middle ear ossicles from the jaw apparatus, presumably due to expansion of the olfactory cortex (Rowe et al., 2011; Rowe and Shepherd, 2015). However, this may not mean a great increase in auditory sensitivity as the cochlea remained short, as in cynodonts. *Hadrocodium* still lacked ossified turbinals (intricate spongy nasal bones that warm and moisten the air as it enters the lungs), and had a primitive chewing and swallowing system. Full ossification of the turbinal bones, that allowed expansion of the olfactory epithelium, is only seen in more advanced crown mammals.

We will now address the evolution of sensory organs and their central projections in early mammals, to provide a picture of their presumed behavior and the selective pressures that were acting on these sensory systems. We propose that olfaction (and touch) played a dominant role in the earliest mammals, participating in linear navigation. In later stages, other senses (together with increasing motor control) contributed to the development of a primitive isocortex containing multimodal, map-like representations of space.

### Adaptations to nocturnal behavior

It is generally agreed that early mammals underwent a “nocturnal bottleneck” (Walls, 1942), which is consistent with the presumed burrowing behavior of cynodonts. However, nocturnal adaptations most likely arose in the ancestral synapsids some 100 million years before the appearance of the earliest mammals (Angielczyk and Schmitz, 2014). Ample comparative evidence supports the nocturnal hypothesis, including the paucity of chromatic receptor cells and visual pigments in the retina, the night-adapted eye morphology (bearing a large cornea to maximize visual input at the expense of acuity, as well as a tapetum lucidum), and the loss of the corneal UV filter in the majority of extant mammals (Heesy and Hall, 2010; Hall et al., 2012; Gerkema et al., 2013). Another character common in nocturnal animals is binocularity, which is assumed to provide benefits in light and contrast sensitivity, but not necessarily in depth perception (Vega-Zuniga et al., 2013). Note that binocularity is concomitant with growth of the direct retino-thalamic visual pathway (as opposed to the alternative visual pathway that relay in the midbrain before reaching the thalamus, which is the more developed in sauropsids), in both birds and mammals (Heesy and Hall, 2010; Gaillard et al., 2013). Noticeably, the chromatine structure of mammalian rod photoreceptors differs between diurnal and nocturnal mammals, with nocturnal mammals displaying a unique inverted pattern with dense heterochromatin condensed in the nucleus' center, and euchromatin in the periphery, while diurnal mammals show the reverse pattern (Solovei et al., 2009). The nocturnal, inverted pattern generates a geometry that works as a collecting lens that maximizes light input into the receptor's light sensitive outer segment.

It is extremely interesting to compare this pattern with non-mammalian species to elucidate a possible evolutionary pattern [note that anthropoid primates are an exception to many of the above adaptations as they have re-evolved color vision (Gerkema et al., 2013)]. In these conditions, other senses, notably olfaction, but also somatosensation and to a lesser extent audition, underwent a compensatory development, which we suggest produced a dramatic change in the lifestyle of the early cynodonts. In later stages, as mammals recolonized diurnal niches, vision (and audition) contributed a significant input to behavioral orientation, perhaps concomitant with expansion of the isocortex.

### Audition and mastication

While the emergence of a tympanic ear took place several times in amphibians, stem reptiles and mammal-like therapsids (Grothe and Pecka, 2014), the emergence of the middle ear ossicles and the consequent amplification of the auditory capacity represent critical acquisitions in mammalian evolution. This process began with a series of modifications of the jaw apparatus in relation to new feeding habits and more elaborate dentition. This condition resulted in the elaboration of the dentary bone in the mandible, which began to make contact with the squamosal bone in the cranium in primitive mammaliamorphs like *Pachygenelus* (a small tritylodont cynodont from the early Jurassic), and gradually replaced the ancestral quadrate-articular jaw joint that is seen in reptiles and mammal-like reptiles (Luo et al., 2001; Luo, 2007). The ancestral quadrate and articular bones became released, and the articular from the lower jaw became the mammalian malleus. The quadrate from the upper jaw evolved into the incus, and attached to the stapes via a stapedial process that appears for the first time in *Brasilitherium* (a Triassic cynodont from Brazil; Luo, 2007). The evolution of the middle ear was a gradual and mosaic process, showing many convergences and divergences in different lineages that began some 200 million years ago (Luo et al., 2001; Luo, 2007). Rowe (1996a,b) reported that detachment of the middle ear ossicles was initially associated with an increase in brain size in fossils like *Triconodon* (a late Jurassic triconodontid, about the size of a domestic cat) and *Hadrocodium* (mentioned above). Subsequent evidence showed that in other early mammals like *Repenomanus* (a carnivore triconodont from the early Cretaceous of China) the reverse is the case: they bear narrow braincases associated with detachment of the middle ear ossicles, indicating that more likely, the acquisition of the middle ear predated the increase in brain size in early mammals (Wang et al., 2001). Another hypothesis is that the volumetric expansion of the olfactory cortex in the lateral hemisphere of *Hadrocodium* forced the detachment of the middle ear ossicles from the jaw articulation (Rowe and Shepherd, 2015). However, the fossil *Yanocodon* (a burrowing triconodont from the early Cretaceous of China) shows increased brain size and ossicles still attached to the mandible (Luo, 2007). Nonetheless, Rowe and Shepherd (2015) interpret this unique fossil specimen as a juvenile, reminiscent of young marsupials, in which the ossicles are attached to the jaw during early development, and are released in later stages.

The elaboration of a coiled cochlea, allowing detection of high frequency sounds (above 10 KHz), took place much later, some 60 million years ago in therian mammals with modern brain sizes (Manley, 2012, 2013). Enlargement of the cochlea occurred concomitant with the expansion of the auditory epithelium or organ of Corti. Furthermore, the elaboration of the cochlea most likely has been accompanied by the acquisition and gradual elaboration of prestin-dependent electromotility of hair cells in therians and placentals, respectively (Liu et al., 2012). As in reptiles, the monotreme auditory epithelium is separated in two components: the basilar papilla and the lagenar macula, while therians have fused both surfaces in the organ of Corti (Fritzsch et al., 2013). More interestingly, although monotremes have an incompletely curved cochlea, it shows some mammalian features like a separation of inner and outer cells in the organ of Corti and nonlinear, cochlear amplification mechanisms that enhance auditory perception (Ashwell et al., 2014). Additional evidence for a mosaic evolution of the inner ear comes from the Jurassic mammal *Dryolestes*, which shows derived features of therian-like auditory cochlear innervation, but still has an uncoiled cochlea (Luo et al., 2011).

Together with increasing auditory discrimination and sensitivity to high frequency sounds, audition may have served a particular role in spatial orientation, by comparing the time differences between auditory inputs to each ear (determining azimuth of the sound source), and especially by processing the variations in the spectral cues associated with movement of the sound source and the direction of the pinnae (for which high frequency detection is relevant). An additional factor are the interaural level differences caused by sound crossing through the head or the body to reach the ear contralateral to the sound source (Heffner and Heffner, 1992a; Grothe and Pecka, 2014). While sauropsids tend to rely mostly on interaural time differences for sound localization, mammals also use frequency analysis to detect source movement and sound distortion processes to obtain spatial information, for which high frequency detection is highly relevant (Grothe and Pecka, 2014). Furthermore, auditory localization is tightly correlated with the control of eye movements (Heffner and Heffner, 1992b).

Although much of sound localization takes place subcortically, it is possible that the auditory cortex plays a role in localization of sound sources as well (Lee and Middlebrooks, 2011). However, it seems that the role of the auditory cortex in sound localization varies across mammals, as auditory cortex ablations tend to have little effect in rats but can have severe effects in cats, dogs, and monkeys (Kelly, 1980; Kelly and Kavanagh, 1986). We still do not know the extent to which auditory development contributed to the initial expansion of the isocortex, but having an efficient system to localize sounds may have provided benefits for establishing multimodal cognitive maps in the cortex and hippocampus.

### The somatosensory system and exploratory behavior

Another sensory system that has been considered relevant for the evolution of the mammalian brain is touch. Mammals have a skin devoid of scales, usually covered by hair and highly innervated with different types of mechanoreceptors. In the region of the mouth, hairs differentiate as sensory vibrissae that move back and forth; furthermore, in some cases the mouth and nose themselves become highly specialized sensorimotor organs. In mammaliamorphs there is evidence of an increased trigeminal sensory input for perioral tactile sensation, but also for mastication and the possible development of muscle spindles and joint receptors that control posture and movement, providing these animals a mammal-like gait (Rowe and Shepherd, 2015). Since there is some evidence of mammal-like skin in mammaliaforms, Rowe and Shepherd (2015) suggested that these animals already displayed a somatosensory or somatomotor cortex in their moderately enlarged brain.

Grant et al. (2013) described a similar “whiskering” behavior in marsupials and rodents. This matches similarities in the anatomical arrangements of the vibrissal musculature, although there was a more elaborate whiskering function in rodents. Unfortunately, extant monotremes bear a derived beak-shaped mouth and may not be useful as models of early mammalian oral behavior. Vibrissae and other somatosensory oral structures can be used in different forms of tactile discrimination (Guic-Robles et al., 1989, 1992), but importantly they also participate in localizing and tracking objects (Krupa et al., 2001; Ahissar and Knutsen, 2008; Knutsen et al., 2008), as well as in orienting behavior (Hartmann and Bower, 2001). In addition to these specializations, increasing forepaw control and dexterity due to the elaboration of descending tracts from the pallium and the eventual elaboration of a corticospinal tract to the brainstem was very likely a positive factor in the generation of exploratory behavior and navigational capacities (Aboitiz and Montiel, 2007, 2012; Rowe and Shepherd, 2015). This points to a cooperative interaction between the oral somatosensory and other sensory and motor systems in spatial orientation in early mammals. Finally, more sensitive skin may have had important effects on the social behavior of early mammals, again in co-evolution with olfactory and pheromonal signals (Porter, 2004; see below). In this context, Lenschow and Brecht (2015) recently demonstrated that social contact evokes a strong anticipatory depolarization and membrane fluctuations in the barrel cortex of rats (representing the sensory input of whiskers), which are different from those seen in free whiskering behavior and are not triggered by non-conspecific stimuli.

### Olfaction in early mammals

Olfaction is the most expansive sense in mammals, with an olfactory receptor gene family that makes up 1% of the mammalian genome, and is some 10-fold larger than in any other vertebrate group (Niimura, 2009; Hoover, 2010). Furthermore, by virtue of their diaphragm-based respiration and the development of a secondary palate in their mouth, mammals are able to actively sniff the air in search of volatile substances, and have accordingly developed the nasal turbinals that facilitated expansion of the olfactory epithelium. This design also allows the generation of retronasal smelling, which combines with taste information from the mouth, producing the complex sense of flavor (Shepherd, 2007; Hoover, 2010). Unfortunately, the effects of this condition in the evolution of taste, and the role of taste in mammalian evolution, are subjects of great interest but there is as yet little comparative evidence. In the neocortex, gustatory representation lies on the insula and the frontal lobe. These regions also process internal sensations and may have benefited from neural expansion in the lateral-frontal pallium, associated with the development of endothermy and more sophisticated homeostatic functions (Smart, 2008).

Returning to olfaction, the early cynodont *Brasilitherium* already displayed partial ossification of the nasal septum and expansion of the posterior nasal cavity, as seen in computerized tomography imaging (Ruf et al., 2014). However, the relative sizes of olfactory structures started a dramatic expansion in mammaliaforms, together with a stepwise amplification of brain size. Rowe et al. (2011) performed X-ray computer tomography on a series of Cretaceous cynodont, mammaliaform and early mammalian skulls, unveiling an association between increasing absolute size of turbinal bones, olfactory bulbs and olfactory cortex on the one hand, and absolute brain size on the other. Rowe et al. (2011) largely attributed this finding to the amplification of olfactive capacity, but also highlighted the elaboration of somatosensory and auditory processing as concomitant factors. One important function served by the olfactory system is olfactory discrimination, both ortho- or retronasal. Shepherd and others have argued that the olfactory bulb is capable of generating an “odor image,” while the olfactory cortex develops “olfactory objects” much like what is found in different stages of visual processing (Shepherd, 2007; Rowe and Shepherd, 2015). Importantly, this implies that the olfactory cortex is functionally equivalent to a higher-order, associative cortical area rather than to a primary sensory area.

### Comparative development of mammalian olfactory cortex

Notably, olfactory and olfactory-related brain regions do not show conservative allometric growth in different species. In mammals, all prosencephalic (anterior brain) components—except limbic structures like the olfactory cortex and hippocampus—follow a general allometric rule in which the growth relations between components are highly constrained, within a two or three-fold range, which may give space for ecological adaptations (Barton et al., 1995; Yopak et al., 2010). On the other hand, olfactory structures and the hippocampus correlate positively between them, but their relative sizes show a general inverse relation with isocortical growth. That is, although they may increase in absolute size, this increase is far more modest than the other brain structures (Reep et al., 2007). Furthermore, Jacobs (2012) claimed that the relative size of olfactory systems depends on the predictability of the food sources in different species. Animals that scan their environment to obtain prey or that have plenty of food available need little navigational capacities and score low in relative size of olfactory components, while species that have to search for their prey tend to score high in olfactory structures. Moreover, it is one thing to find one's source of food, it is another to capture it. Thus, animals like carnivores, which have to develop complex strategies to chase their prey, display both relatively large olfactory structures and a large isocortex, while simians that have no difficulty in obtaining food but have a complex, visually oriented social life have a relatively small olfactory system and a large isocortex (see also Gilad et al., 2004). On the other hand, microbats have both a small olfactory system and a small neocortex, while prosimians and insectivores have large olfactory components and a small isocortex in relation to total brain size. Jacobs (2012) asserts that prosimians and insectivores, with a large olfactory cortex and a small isocortex, better resemble the condition of ancestral mammals.

### Olfaction in social and maternal behavior

Social and maternal behaviors were also significant innovations in early mammalian evolution, and deserve to be discussed in this context. Considering that early mammals had nocturnal habits and a significant reduction of the visual system, a large part of their social signaling system may have relied on olfactory and pheromone cues. Chemical signals are involved in territorial marking, individual identification and sexual behavior, among other functions, which may have been quite important for early mammalian behavior. The olfactory system in mammals is importantly connected to areas involved in social reward, modulating neuroendocrine functions that facilitate social learning, and maternal behavior (Broad et al., 2006; Sanchez-Andrade and Kendrick, 2009). The accessory olfactory bulb can detect chemical signals like proteins of the major histocompatibility complex or urinary proteins that not only permit recognizing individuals, but also their genetic relatedness (Brennan and Kendrick, 2006). Interestingly, mammalian adult olfactory neurogenesis in rats has been found to depend on reproductive behavior (Feierstein, 2012). Furthermore, the social behavior modulators oxytocin and vasopressin are expressed in the main and accessory olfactory bulbs and participate in the formation of short-term social odor memories (Wacker and Ludwig, 2012).

Pheromones and olfactory cues may be critical for mammalian maternal behavior through modulation of the neuroendocrine system (Lévy et al., 2004; Sanchez-Andrade and Kendrick, 2009; Schaal, 2010). Milk secretion is triggered by oxytocin in response to the sight, sound and smell of human babies (Leng et al., 2005). While not lactating, female rodents find the odor of pups aversive, while after parturition and during lactation, the same stimulus results in a potent approaching trigger. Furthermore, in rodents mother-child individual recognition seems to depend exclusively on the main olfactory system, and the main olfactory bulb undergoes profound synaptic changes with exposure to offspring odors at parturition, contributing to the memorization of odors and long-term maternal behavior (Lévy et al., 2004). Olfaction is perhaps more important to puppies, who have to search for the milk sources in their mother's bellies. In therian mammals, chemical signals emanating from mammary glands are key for arousal, attraction and orienting behavior to the mother and to reach the milk sources (Raihani et al., 2009; Stevenson, 2009; Schaal, 2010). For example, rabbit kittens have shown specific nipple-search behavior in response to cues like the mammary pheromone (Schaal et al., 2003; Raihani et al., 2009). This may be considered as the earliest navigational function of olfaction in a newborn mammal. In addition, milk and belly secretions may have served as the first social signals secreted by early mammals, as abdominal odor cues are used by newborn mammals to distinguish between different adult conspecifics (Schaal et al., 2009).

Nonetheless, monotremes did not concentrate mammary glands and teats. Milk is produced from secretory glands located in their bellies, and this condition may be closer to that of early mammals. Moreover, monotremes start producing milk just after they lay the eggs, and not at the time newborns hatch (Enjapoori et al., 2014). One hypothesis claims that milk evolved from belly secretions that originally served to avoid dessication of the soft-shelled eggs laid by primitive mammals, resembling the condition of modern monotremes (Warren et al., 2008). Furthermore, monotreme milk contains antibacterial proteins that help protect the altricial newborns from pathogens (Enjapoori et al., 2014). Interestingly, the nutritious protein component of the milk, casein, is already present in the milk of monotremes, but these animals also express vitellogenin in their eggs to nourish the embryos before hatching. In therians, however, vitellogenin genes have been pseudogenized (inactivated by nonsense mutations) at the expense of evolving more copies of casein genes (Brawand et al., 2008). In living monotremes the olfactory system has been reported not to be functional at birth (Ashwell, 2012), perhaps implying that the active compound eliciting preferential orientation to milk sources is associated with the acquisition of nipples in therian mammals, or that extant monotremes lost this capacity in their evolution. The latter is plausible considering that they have a poorly developed olfactory system, perhaps at the expense of an increased electrosensory sensitivity that aids them in searching for food (Ashwell, 2013). Whether early mammals relied on navigational cues to find their mother and the milk sources is still unknown, but one can provisionally speculate that such was the case.

### Olfactory connections with the hippocampus and role in navigation

**Figure 2:**
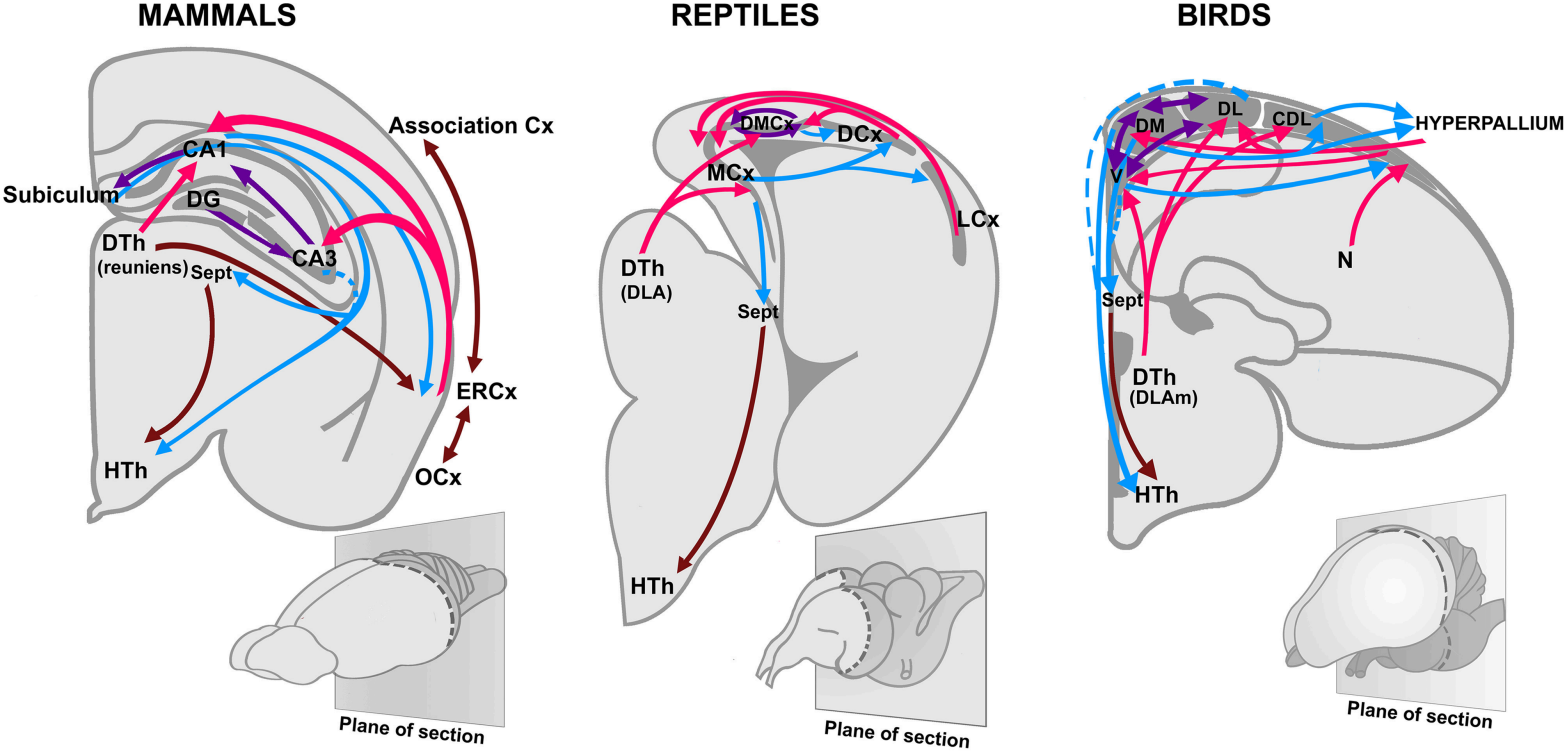
**Olfactory projections to the hippocampus in birds, reptiles, and mammals**. Based on Striedter (2015), with permission. CA1, *Cornu Ammonis 1*; CA3, *Cornu Ammonis 3*; Cx, Cortex; DG, Dentate gyrus; DLA, Dorsolateral anterior nucleus; DLAm, medial part of the dorsolateral anterior nucleus; DTh, Dorsal thalamus; HTh, Hypothalamus; ERCx, Entorhinal cortex; DCx, Dorsal cortex; DL, dorsolateral division of the hippocampus; DM, dorsomedial division of the hippocampus; DMCx, Dorsomedial cortex; LCx, Lateral cortex; N, Nidopallium; MCx, Medial cortex; OCx, Olfactory cortex; Sept, Septum; V, ventral division of the hippocampus (also named V-shaped area).

**Figure 3:**
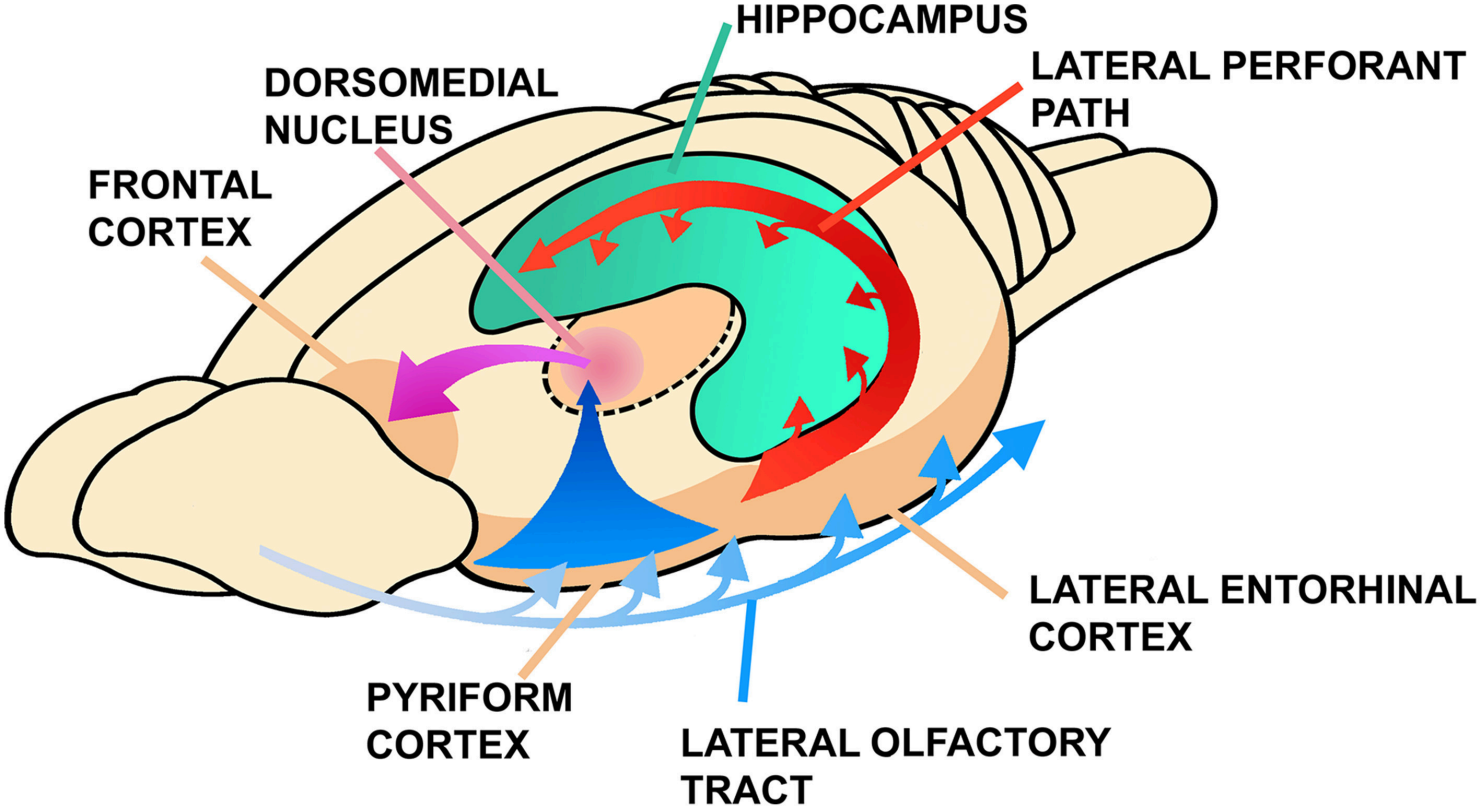
**Connections between the olfactory cortex, the hippocampus and the frontal cortex in mammals**. Note the thalamic olfactory projection that is relayed to the frontal cortex. Modified from Lynch (1986), Figure 8A, pp. 28, ©1986 Massachusetts Institute of Technology, by permission of The MIT Press.

Jacobs (2012) recently argued that more than as a sense involved in discriminating stimuli, olfaction works as a reference system for spatial navigation, guiding the animal to locate food sources, or mates (see also Eichenbaum, 1998). Furthermore, Jacobs (2012) suggested that the navigational properties of the olfactory system serve as scaffolding for the evolution of a parallel orientation map in the hippocampus. Despite some variations, a hippocampal region associated with spatial orientation (Day et al., 1999, 2001; Rodríguez et al., 2002), and an important olfactory-hippocampal projection (Striedter, 2015; Figure 2), are conserved features of all amniotes. In rodents, the olfactory system connects with the hippocampus through the entorhinal cortex, which also forms extensive associative networks with other sensory modalities in the isocortex (Lynch, 1986; Haberly, 1990; Figure 3).

Hippocampal cells display a fine olfactory recognition capacity, and spatial and non-spatial (olfactory) responses segregate in alternating bands in the hippocampal region (Hampson et al., 1999). According to Eichenbaum (1998), more than participating in sensory discrimination, the hippocampus is critical for associating relationships between different odors and for associating odors with different situations. In this line, Vanderwolf (2001) observed in the rat strong oscillatory gamma activity in the hippocampus, associated with active sniffing or by blowing odorants with a cannula under anesthesia, but not by stimulating other modalities in absence of movements. In line with our argument, Vanderwolf proposed that the hippocampus is basically an olfactory-motor system and the cognitive functions of this structure are a secondary acquisition. Furthermore, hippocampal “time cells” have been described that codify sequences of events that contribute to the formation of a spatio-temporal representation of the environment (MacDonald et al., 2011; Eichenbaum, 2014; Davachi and DuBrow, 2015). These representations are relayed to the prefrontal cortex to assimilate new memories in contextual networks in the process of memory consolidation (Preston and Eichenbaum, 2013).

Nonetheless, a more widespread interpretation of hippocampal function is that it contributes to establishing a Cartesian map of the space in which the animal navigates. Crucial elements for this function are the so-called “place cells” located in the hippocampal CA1 region, and the “grid cells” in the entorhinal cortex (Alme et al., 2014; Krupic et al., 2015). Particularly, the grid cells of the entorhinal cortex generate a bidimensional, grid-like periodic pattern as the animal moves in space (Fyhn et al., 2004; Hafting et al., 2005) and seem to be critical for spatial orientation. During navigation, information must be integrated between egocentric external cues and allocentric updates of current actions and position. Both kinds of signals are required to generate a time-independent, two-dimensional map during spatial exploration, provided by hippocampal place cells (Buzsáki, 2005). Particularly relevant in this context is information about head direction signals, conveying information from vestibular and motor systems, which provide a directional axis during exploration (Taube, 2007). These two types of input (external cues and body position and movement) are segregated in different regions of the entorhinal cortex, the dorsolateral entorhinal cortex providing information about external sensory cues, and the ventromedial entorhinal cortex conveying information about self-position and motion (Fyhn et al., 2004; Lisman, 2007). While the classical view is that entorhinal grid cells preferentially code information about the animal's self-motion (propioception), some evidence indicates that in some conditions, navigation is possible using only sensory landmarks (Poucet et al., 2013) These authors propose that bodily information is crucial for navigation, particularly in the dark, where grid cells are driven by self-motion inputs and in turn grid cells drive hippocampal place cells (Poucet et al., 2013).

Lastly, it is worth mentioning that the main regions in which adult neurogenesis occurs in mammals are precisely the olfactory bulb (Sanai et al., 2011) and the dentate gyrus of the hippocampus (Song et al., 2012). In this context, Sahay et al. (2011) have proposed that, despite their apparent differences, new granule cells in both structures reflect an adaptive mechanism to encode contextual information by modulating the process of pattern separation, that is, dissociating similar inputs on the basis of contextual information.

### The isocortex as a multimodal interface

Early mammals were probably nocturnal and fossorial, a condition in which internal cues like propioceptive information, together with sensory information from the olfactory and the somatosensory systems were crucial for linear navigation. In these conditions, orientation may have relied mainly on one-dimensional maps, coding for sequences of events in a time series (Buzsáki, 2005; Eichenbaum, 2014). However, as early mammals started to diversify and invaded diurnal niches, additional sensory inputs like vision and hearing, providing more detailed information on spatial relations, became increasingly important for generating accurate bidimensional and time-independent spatial maps.

Noticeably, the reptilian dorsal cortex (presumably a field homolog to the mammalian isocortex) can be compared in function and structure with the entorhinal and subicular cortices of mammals, which connect cortical and limbic areas with the hippocampus (Powers, 1990; Aboitiz et al., 2003; Aboitiz and Montiel, 2007; Butler et al., 2011). In this context, the early isocortex may have differentiated from the ancestral dorsal pallium (dorsal cortex in reptiles), as an interface between the olfactory cortex and the hippocampus to provide additional sensory information involved in navigation, perhaps initially somatosensory (Aboitiz et al., 2003; Aboitiz and Montiel, 2007). Supporting this proposal, the early mammaliaform *Castorocauda* may have had a rudimentary somatosensory cortex (Rowe et al., 2011), contributing to orientation behavior. Even if the reptilian dorsal cortex receives visual input, it does not participate in vision but rather in learning and memory (Powers, 1990). This may have been the case in early mammals, especially considering their nocturnal, burrowing habits. At later stages, the early expansion of the somatosensory region provided a substrate for the eventual strengthening of additional inputs like vision, especially when animals invaded diurnal niches.

In its origins, the expanding dorsal pallium of cynodonts and mammaliaforms displayed a predominantly tangential organization, with afferents running in the superficial marginal zone and contacting the apical dendrites of pyramidal neurons, as observed in the dorsal cortex of reptiles and the olfactory cortex of mammals and reptiles (Fournier et al., 2015; Naumann et al., 2015). This resembles the anatomical disposition of cortical afferents in small-brained, extant mammals (Nieuwenhuys, 1994). Furthermore, the neocortex has been claimed to display an intrinsic tangential organization, reminiscent of that of the olfactory cortex (for more details, see Shepherd, 2007, 2011; Bosman and Aboitiz, 2015; Fournier et al., 2015; Naumann et al., 2015). The radial organization of the modern isocortex was acquired later in crown mammals or their direct ancestors, concomitant with the differentiation of primary sensory areas. There were several embryonic mechanisms involved in the generation of a radial arrangement, including the development of an embryonic subplate, the amplification of reelin signaling, and the differentiation of a proliferative subventricular zone in the developing neuroepithelium (reviewed in Aboitiz et al., 2003; Aboitiz and Montiel, 2007; Montiel and Aboitiz, 2015; see also Abe et al., 2015). Thus, in its origins, the rudimentary isocortex was “imprinted” by the tangential and laminar architecture of the olfactory cortex and hippocampus, and became modified into a radial design only at later stages. This view is consistent with Sanides' original notion that the cerebral cortex initially developed by the expansion of peri-allocortical regions, which serve as an interface between the neocortex and limbic cortices (Sanides, 1968).

In the common ancestor of crown mammals, the isocortex was fully developed and had a conspicuous radial organization, with four visual areas, four somatosensory areas, a gustatory or insular region, and an auditory area (Kaas, 2013). In addition, there were medial and orbitofrontal cortices rostrally and a cingulate cortex medially (Kaas, 2013). More recently, a posterior parietal and a multimodal area were also described in the opossum, suggesting that somatosensory and multimodal regions were also important in the isocortex of primitive mammals (Dooley et al., 2015). Analyzing the phylogenetic distribution of the gyrencephaly index (the degree of convolutedness) across 102 mammalian species, Lewitus et al. (2014) concluded that the common ancestor of crown mammals was most likely gyrencephalic. This implies that the small, lissencephalic brains of some species like insectivores should be viewed as derived, simplified forms, or alternatively that gyrification took place many times in modern mammals.

## Discussion

Brain size has increased significantly only in mammals and birds. In both cases, the increase in exploratory behavior propelled by homeothermy, benefited from increased telencephalic, associative networks conveying multimodal information about spatial relations. In mammals, early brain expansion was associated with olfactory navigation (together with somatosensory and propioceptive information), while sauropsids relayed mainly on vision. This difference established a diverging point after which mammals and birds underwent separate evolutionary trajectories, while conserving basic functional mechanisms of neural processing (Aboitiz and Montiel, 2012; Montiel and Molnár, 2013).

In the Mesozoic, early mammals were a successful clade that radiated, together with the diversification of flowering plants and the insects that coevolved with them (Aboitiz and Montiel, 2012). Nonetheless, the ecological niche that early mammals constructed was characterized by some key features like a burrowing, nocturnal way of life, and mothering behavior, together with the secretion of milk. The masticatory apparatus underwent dramatic rearrangements and liberated the ancestral jaw articulation to eventually become co-opted for impedance amplification in the middle ear ossicles. Furthermore, the loss of scales and increasing tactile behavior, particularly in the front of the mouth, together with the development of tactile vibrissae, facilitated exploratory behavior and were important in subterranean burrows.

Although we now know that Mesozoic mammals exploited a variety of ecological options, we can speculate that increasing somatosensory sensitivity in the mouth, the elaboration of direct motor cortical control of the forepaws (which is more pronounced in digging mammals; Heffner and Masterton, 1983), the development of eyelids and even possibly the loss of scales (for example the naked mole rat; Dhouailly, 2009) may be consequences of another early, burrowing “bottleneck” beside the nocturnal adaptations, that yielded profound modifications in these animals. In these conditions early mammaliaforms may have relied on predominantly olfactory information to orient themselves. The olfactory-hippocampal axis has been proposed as a key network for orienting behavior and spatial navigation (Jacobs, 2012), which, while it may be relevant in most vertebrates, was critical for the behavior of the earliest mammals. Additionally, a critical component at this point was somatosensation, particularly in the orofacial region, which projects to the dorsal pallium where the isocortex emerged. Furthermore, moderately increasing auditory sensitivity may have been relevant in subterranean life, especially for social communication. However, the major expansion of the isocortex took place in later stages, when other senses (like vision and audition) began to provide information to the hippocampus to generate multimodal, bidimensional orientation maps.

Our hypothesis has common ground with those proposed by Lynch (1986), Rowe et al. (2011), and Rowe and Shepherd (2015) in that olfactory systems were key in early mammal evolution. We add to these hypotheses the role of the emergent isocortex as a multimodal interface in the olfactory-hippocampal axis for behavioral navigation. In primitive cynodonts, orientation was based on sequential time series based on olfactory, tactile and proprioceptive cues. Expansion of the isocortex was associated with the inclusion of other sensory modalities like vision and audition, yielding bidimensional orientation maps of space rather than a linear representation of items.

Finally, we highlight the argument that brain evolution cannot be fully understood through developmental, anatomical, functional, or behavioral perspectives alone. This is because we need to combine such approaches to reach a comprehensive understanding of the genetic and epigenetic mechanisms generating developmental variability, in concert with the selective pressures exerted by the ecological and behavioral conditions animals face to successfully reproduce. Given this background, brain evolution is subject to conserved processes to which contingent adaptations are added, that may leave enduring marks in subsequent evolutionary modifications (like isocortical lamination). On the other hand, there are also conserved requirements for proper brain function and for the generation of complex perception and behavior that shape circuit and network architecture in similar ways in different lineages.

### Conflict of interest statement

The authors declare that the research was conducted in the absence of any commercial or financial relationships that could be construed as a potential conflict of interest.
